# Structural predictors and latent maturity regimes of robotic readiness in global health systems: evidence from machine learning-based latent clustering and class prediction

**DOI:** 10.3389/frobt.2026.1835473

**Published:** 2026-07-16

**Authors:** Moumita Mukherjee, Raja Hashim Ali

**Affiliations:** 1 Institute of International Health, Charité - Universitätsmedizin, Berlin, Germany; 2 Digital Business and Data Science, Department of Business, University of Europe for Applied Sciences, Potsdam, Brandenburg, Germany

**Keywords:** clustering, digital health maturity, latent readiness regimes, machine learning, recoverability, robotic readiness

## Abstract

**Background:**

The systematic integration of robotics into health service delivery systems requires periodic assessment of robotic readiness in terms of digital-health maturity regimes across countries. The current study aims to cluster 169 countries into maturity regimes and classify and predict cluster membership accuracy based on digital-health maturity dimensions determining the system’s perception and interoperability, coordination, and workforce–regulatory reliability readiness. These country-level proxy concepts are applied due to a dearth of cross-country robotic readiness measures at the global level. The study also proposed an adaptive readiness framework for robotic deployment decision-making.

**Methods:**

This study applies a multilayered robotic systems readiness proxy framework based on diffusion of innovations and multi-agent systems theories, which hypothesise health-system readiness as adaptive and learning-driven instead of static. Using 31 global digital-health maturity indicators from the Global Digital Health Monitor 2023, three latent proxy dimensions are constructed to measure perception/interoperability readiness, governance/coordination readiness, and workforce–regulatory reliability readiness. The data represent 169 countries at different stages of digital-health maturity, starting from phase 1 to phase 5, where 67 countries reflect evidence-based digital-health maturity status and 102 countries had no data reported and are below phase 1. The sample included these 102 countries in the study, imputing the lowest values for each indicator to prioritise them and eliminate participation bias in global health policy decisions. To address concerns regarding missing data and its handling, the analysis was repeated across different robust scenarios. The latent proxy dimensions are created using principal component analysis, reducing 31 dimensions to 3 dimensions. Their associations were explored using ordinary least squares regression. Unsupervised clustering techniques (K-means, agglomerative hierarchical clustering, and Gaussian mixture modelling) were applied to identify latent readiness regimes, and comparative model assessment brought a four-cluster solution. External validation was conducted against the World Bank classification of countries. The separability of the latent regimes was assessed through repeated cross-validations and post-clustering recoverability analysis using conservative supervised learning models, where five latent clusters were considered in line with the World Health Organization’s five-phase maturity categories.

**Results:**

The three proxy indices have shown internally coherent loading structures with 85%–95% scale reliability. Significant positive structural associations are evident where the perception/interoperability (β = 0.343, p < 0.01; observed-only scenario) and governance/coordination readiness (β = 0.838, p < 0.01; baseline scenario) layers reflect stronger partial associations with workforce–regulatory reliability readiness (R2 = 0.892, p < 0.001; baseline scenario) for intelligent robot implementation in healthcare. K-means, Gaussian mixture modelling (GMM), and agglomerative (hierarchical) unsupervised learning models reveal good cluster distinctiveness (silhouette = 0.633 [k = 4]; 0.617 [k = 5]) varying between the scenarios, ranging from 0.357 to 0.514 under four robust cluster regimes, indicating distinct maturity regimes in the “RPI–MRCI–ACRI readiness” space. Robustness analyses indicated that the readiness structure remained considerably identifiable across three alternative missing data scenarios, although some boundary cases were overlapping. External validation using the World Bank and WHO’s country classification indicated profound alignment with the final cluster regimes. Post-clustering recoverability analysis depicted that the latent clusters were significantly separable within the readiness space. Cluster membership in moderate to very good system readiness requires ∼30% contribution of each of the readiness indices to classify the country at the advanced to transformed phase of intelligent robotics implementation readiness in most of the scenarios under K-means and GMM. Machine learning (ML) models are run on a five-cluster specification given the main data categorisation requirement of phase “1” to phase “5.” Among four supervised machine learning models, random forest and XGBoost achieved the highest (accuracy up to 0.980; area under the receiver operating characteristic curve (AUROC) ≈ 1.00) and most reliable performance (cross-validated accuracy ≈ 0.91), supporting robust separability and recoverability of the latent readiness regimes. SHAP analysis identified macro-level governance and coordination readiness index (0.111) as the most influential predictor of cluster classification under K-means and perception readiness (0.106) as the most influential under GMM.

**Conclusion:**

Findings uncover heterogeneous readiness clusters of countries and exhibit that digital health governance coherence, standards and interoperability, and institutional digital literacy are stronger predictors of robotics integration potential in healthcare than technological dimensions alone. The study does not provide a direct estimate of robotics deployment but offers a thoughtful, system-level framework for comparing structural enablers relevant to robotics ingestion for health-system strengthening. An adaptive monitoring framework is proposed as a conceptual future-work direction rather than an empirically validated component of the present study.

## Introduction

Rapid and growing use of intelligent robots in healthcare has shown increased health-system performance with greater robotic sensory perception enhancing health systems’ time and cost efficiency (Li et al., 2024; Agarwal et al., 2020). The utility of intelligent robots in critical care is widely acknowledged, saving patients as well as healthcare personnel and reducing the risk of infections while rendering service during COVID-19 (Burki, 2021; Fong et al., 2021). Robotics is defined by the US National Library of Medicine as “the application of electronic, computerised control systems to mechanical devices designed to perform human functions” (Geogopoulos et al., 1987; Li et al., 2024), where artificial intelligence (AI)-driven intelligent robots, including mechanoid, humanoid, android, and animalistic robots, can reason and learn to enhance decision-making (Huang et al., 2023; McKendrick et al., 2021). Simultaneously, the successful robotics integration into health service delivery varies not only due to the technical efficiency of robotic systems but also because of the maturity of the broader health-system environment in which those systems are expected to operate. Availability of operational digital infrastructure, interoperability, strategic capacity, workforce preparedness, data ethics, and regulatory conditions are the broader system contexts that are likely to shape whether robotics can be initiated, synchronised, governed, and sustained in regular healthcare practice.

### Robotics in healthcare

As evident, existing healthcare robotics research has largely focused on technical layout, clinical applications, human–robot interactions, and implementation challenges within specific settings. A systematic review of 94 research studies categorised the determinants of intelligent robot use at technological, organisational, and individual levels (Huang et al., 2023). Another study exploring ways to improve robots’ enquiry processing ability applied a hybrid intelligent computing model to improve robotic sensory perception, related decision-making, and performance in human–robot interactions using self-organised computing (Albekairi et al., 2025). One study examined sensors, basic robotic algorithms, and autonomous navigation technologies used to perform patient-following by robots and diagnostic assistants (Shin et al., 2025). A qualitative descriptive study based on diffusion of innovations theory was conducted to understand collaborative robot (cobot) implementation in critical care settings and found that human dependence hinders cobot efficacy (Birkhoff et al., 2024). A major challenge in comparative global analysis on robotics implementation in healthcare across countries is that direct country-level measures of healthcare robotics readiness are scarce. Widely available international datasets rarely report standardised indicators of robotic deployment, robotic service density, or robotics-specific implementation maturity. Furthermore, limited attention has been given to exploring the association between macro-level digital-health maturity and structural readiness conditions required for the future integration of robotics at the national, regional, or global levels. A scoping review exploring the utility of healthcare robotics beyond surgical and rehabilitation environments emphasised that infrastructural readiness with robust coordination mechanisms is crucial to enhance robots’ performance efficiency (Morgan et al., 2022).

### Digital health capacity and readiness

Given this context, digital health system readiness is crucial in every healthcare setting and is often assessed through the maturity of leadership and governance, interoperable systems and supporting infrastructure, and the preparedness of trained healthcare professionals to adopt the systems’ readiness in adopting digital health tools efficiently and effectively (World Health Organization, 2024). Researchers applied multidomain models to explore how digital health readiness in healthcare settings influences the adoption of digital health tools and how the presence of digital health maturity (DHM) gaps limits the adoption and scalability of digital health implementation (Bober et al., 2024; Alotaibi et al., 2025).

Acknowledging the need, several studies assessed digital health systems readiness and measured the capacity to adapt to rapidly evolving digital health interventions that are shaping the future of health systems. A mixed-method review of 21 studies identified the lack of workforce capacity and infrastructure as major barriers to digital capability readiness in healthcare (Alotaibi et al., 2025). Integrating specialised training with organisational support and infrastructure would increase digital readiness and capability of healthcare professionals (Alotaibi et al., 2025). Navigating through a rapidly evolving digital health landscape, the healthcare system and its diverse healthcare professionals are facing significant challenges to adapt to new technologies for bringing effective outcomes, and high-end intelligent healthcare robotics is one of them (Huang et al., 2023; Nazeha et al., 2020). A bibliometric review of 287 systematised studies explored the barriers and facilitators influencing readiness, where transformation with the Internet of Things, mHealth application, and telemedicine is reshaping healthcare in favour of patient-centric service (Stoumpos et al., 2023). A scoping review of 65 articles concludes that digital health ecosystem readiness with adaptable and interoperable digital tools, foundational information and communications technology infrastructure, and a mature enabling environment is crucial in improving access and quality of primary care (Erku et al., 2023). Such ecosystem readiness becomes critical further with the conceptual shift of healthcare towards P5 medicine—predictive, personalised, preventive, participatory, and precision—suggesting that intelligent healthcare robotics should be leveraged to support diagnosis and treatment-related decision-making (Denecke and Baudoin, 2022).

Given this backdrop, system readiness is to some extent evident in health facilities with adequate investment and digitally literate staff, leading to lean coordination in service delivery resulting in higher patient satisfaction (LeFevre et al., 2017). Another review of 31 studies assessed gaps in existing digital-health maturity models and proposed a digital health communication maturity model, as most previous models focused more on infrastructure and less on user engagement—reflecting weaker readiness (Kim and Namkoong, 2025). Among different digital health interventions, the application of intelligent robots is gaining significant importance to provide better services with optimum sensory power, reliable autonomous coordination, and less human assistance in delivering care services (Huang et al., 2023).

Although digital transformation and robot applications are expanding, there is limited research on structural determinants, where most studies focus on technical aspects of robot operations. However, to ensure technical efficacy in healthcare robotics, assessment of the digital health capacity of the national healthcare system is fundamental, which conditions a system’s readiness for perception and interoperability (RPI), enables macro-level readiness for governance and coordination (MRCI), and supports Autonomous Capability and regulatory Reliability Index (ACRI). The background research reflects the need for a structured, maturity-based investigation to explore the diffusion of robotic innovation phases and identify systemic enablers and barriers associated with its technical maturity. The current study hypothesises that countries with uneven digital systems, absence of focused leadership, weak interoperability, limited governance mechanisms, lack of digital literacy in the workforce, or inadequate regulatory safeguards may face greater challenges to ideate, adopt, and scale robotics-supported healthcare services. Thus, digital-health maturity may serve as a crucial system-level prerequisite to generate matured enabling environment for healthcare robotics. In this backdrop, the current research has explored the following research questions to improve policy targeting.

## Research questions

RQ1 (construction and structure):

How can digital-health maturity dimensions be systematically reduced into three interpretable proxy dimensions reflecting perception/interoperability readiness, governance/coordination readiness, and workforce–regulatory reliability, and how are these dimensions structurally associated at the country level?

RQ2 (clustering and robustness):

Can countries be grouped into distinct latent readiness regimes based on the above-mentioned three proxy dimensions, and does a four-cluster solution provide a robust and interpretable structure across alternative missing data handling scenarios?

RQ3 (validation and recoverability):

To what extent are the identified latent regimes externally valid and structurally stable, based on the alignment with established country groupings and recoverability under a post-clustering classification framework?

**FIGURE 1:**
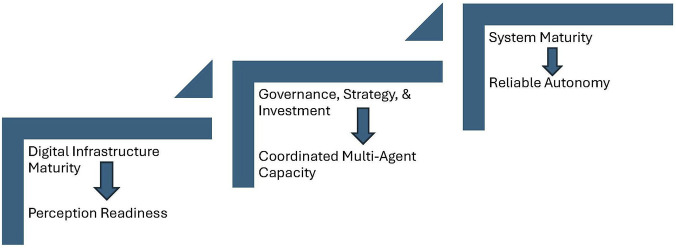
Analytical framework for robotic system readiness. Source: created by the authors.

The robotic readiness analytical framework is created following two theoretical models, which are presented in Figure 1. The non-linear phased progression in readiness across RPI, MRCI, and ACRI is based on the diffusion of innovations theory, pioneered by Rogers et al. (2014), where digital infrastructural architecture and the degree of interoperability operate as innovation attributes that condition health system–level adoption. In this lens, RPI is built with the system’s knowledge and persuasion (stages 1 and 2 of diffusion) on the need for infrastructure, interoperability, and data management to create a positive enabling environment that conditions robotic sensing capacity. MRCI and ACRI are considered as related to the decision, implementation, and confirmation phases of diffusion (stages 3–5), shaped by multiagent-led coordinated governance and strategic institutional investment focusing on health professionals’ digital literacy under a digital regulatory framework. Furthermore, the multi-agent systems theory from distributed AI research (Sifakis, 2019) is considered to capture the layered systemic dependencies required to ensure operational smoothness among robots + digital systems + human actors. It is hypothesised that a multi-agent action framework will create an enabling mature environment for intelligent robotic innovation (sensing + coordination + autonomy) in alignment with structurally stable and rule-governed digital architecture. Together, these frameworks justify the empirical sequencing from principal component analysis (PCA) to ordinary least squares (OLS), clustering to recoverability. The analytical framework visualises that interoperable infrastructure (RPI) forms the first-order condition for coordinated governance architecture (MRCI), which in turn becomes a sufficient systemic enabler with workforce capability under a trustworthy ethical regulatory framework (ACRI). Such theoretical anchoring integrates sociotechnical diffusion logic within the systems-engineering hierarchy to anchor global robotic readiness maturity regimes created by applying machine learning–based clustering and recoverability. This model shows a plausible pathway to create an enabling environment for intelligent healthcare robotics comprising sensing capacity, coordination capability, and accurate, safe, and precise autonomous decision-making with less human assistance (Líšková et al., 2025; Huber Giron-Nieto et al., 2025; Sifakis, 2019).

Layer 1: H1: RPI is created using dimensions of standards and interoperability, infrastructure, and services and applications, conceptualising the higher DHM with the availability of interoperable health and hospital information systems and uniquely identifiable information of facilities, patients, and administrators integrated within scalable infrastructure (including equipment—computers/tablets/phones, supplies, software, devices, etc.) under efficient network readiness improves the system’s perception, thereby creating an environment for improved robotics implementation capacity.

Layer 2: MRCI is created using two pillars—leadership and governance and strategy and investment. Macro-level governance and coordination requires robust shared-task coordination and conflict-resolution mechanisms following governance protocols through the planned integration of strategic leadership, funding structure, and planned institutional alignment.

H2.1 The second layer, therefore, conceptualises that a better presence of digital health governance mechanisms, digital transformation planning with equitable technology adoption strategy, and structured public–private investment creates a systemic environment for macro-level coordination capacity.

H2.2 Efficacy of MRCI increases when perception and interoperability readiness are in place.

Layer 3: Consistent and reliable autonomous decision-making requires a clear regulatory framework on data security and privacy and a trained (before and after deployment of intelligent robotic systems) workforce to monitor the adherence mechanism and regulate or certify the services even during uncertainty.

H3.1 The third layer hypothesises that higher systemic reliability enables health professionals with their autonomous decision-making capacity to deploy intelligent robots in healthcare service delivery.

H3.2 Efficacy of ACRI increases with higher levels of perception, interoperability, macro-level governance, and coordination mechanism in the system.

## Methods

### Workflow

**FIGURE 2:**
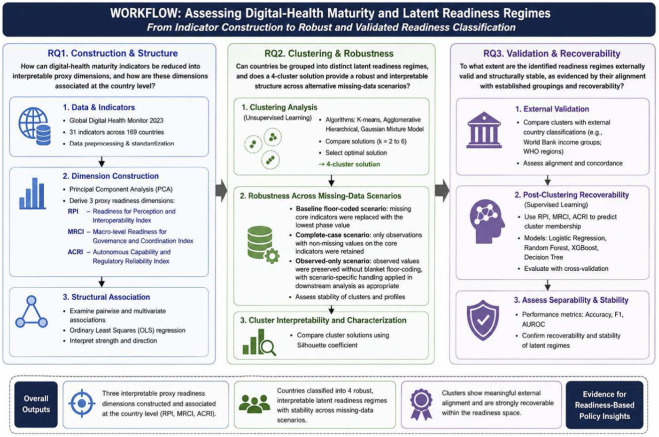
Workflow diagram. Source: created by the authors using GPT 5.0.

All the preprocessing, clustering, validation, robustness, and recoverability analyses were conducted in a reproducible Python/Jupyter workflow. The workflow depicts the whole analytical flow with reference to each research question, ensuring transparent data coding, explicit handling of missing data scenarios, and reproducible generation of tables and figures in the background. The workflow is depicted in Figure 2.

### Data source and study design

The study adopts a multilayer analytical framework to explore digital-health maturity at the country level, measuring structural readiness fitting for future robotics ingestion. It used data from the WHO Global Digital Health Monitor (GDHM) 2023, covering diverse system-level indicators spanning the leadership and governance; strategy and investment; policy, legislation, and compliance; workforce; standards and interoperability; Infrastructure; and services and application domains. GDHM is an interactive dashboard reflecting the status of 169 countries across the world in prioritising and monitoring digital health ecosystems. GDHM measures progress in seven pillars to enhance digital health system readiness through identifying financial and technical assistance needs, integrating each pillar with key digital health implementers. In line with such an initiative, the current research constructed three proxy system readiness indices.

### Variable structure and proxy readiness framing

The original GDHM indicators are not direct robotics-adoption measures; thus, the constructed indices are proxy readiness dimensions created from digital-health maturity indicators, which may plausibly fashion positive conditions for future robotics ingestion. The three latent dimensions are constructed as follows:

1. Perception and interoperability readiness (RPI)
2. Macro-level governance and coordination readiness (MRCI)
3. Autonomous capability and regulatory reliability (ACRI)

The dimensions preserve the layered logic of the analytical framework and clarify that the measures reflect structural digital-health maturity proxies crucial to robotics integration in health systems and not direct robotics-specific performance variables. The variable description tables (Supplementary Tables 1–3) illustrate the variables used to construct the three readiness indicators.

### Data preprocessing and missing data scenarios

The study used the Excel source file in Python to apply explicit recoding of maturity-phase values into ordered numerical scales and standardised them to ensure comparability across indicators. To ensure methodological transparency in missing value treatment, observed values were mapped to ordered phase levels, whereas non-observed ones—countries with no reported maturity information—were preserved explicitly at the preprocessing stage without collapse to support later robustness checks. From a policy relevance perspective, including countries with no reported data helps identify the transition in each annual survey and measure the progress in DHM globally, focusing on long-term global digital-health maturity gain. For robust handling of missing data, the analysis was repeated under multiple scenarios:

1. Baseline floor-coded scenario: missing indicator classes presented as no reported data were replaced with the lowest phase value.
2. Complete-case scenario: only observations with non-missing values on the indicators were retained.
3. Observed-only scenario: observed values were preserved without blanket floor-coding, with scenario-specific handling applied in downstream analysis as appropriate.

The scenarios were compared to assess whether index structure, regression patterns, and cluster solutions related findings were stable across different scenarios.

### Construction of proxy indices using principal component analysis

PCA was applied to construct three composite readiness indices (RPI, MRCI, and ACRI), conceptually defined from seven pillars of DHM indicators, ensuring robust dimensionality reduction. These indicators measure the DHM status of each country with respect to any indicator in five different phases: from phase 1 to phase 5—emerging, developing, defined/established, advanced/integrated, and optimised/transformational. The first principal component of each of the three PCA models is defined as ${PC}_{1}=\sum_{j=1}^{p}w_{j}\;x_{j}$ .

Operationalised as PC1=w1X1+w2X2+⋯+wjXj (1)

where *X*_*j*_ denotes the *j* number of DHM domain indicators and *w*_*j*_ from 1 to *j* are eigenvector weights derived from the correlation matrix.

The first component (PC1) maximises the explained variance and is thus retained as the index score values (Equation 1). For each index, the first principal component was retained as the resultant index. The resulting indices—RPI, MRCI, and ACRI—were directionally consistent such that higher values consistently reflect higher degrees of readiness. The standard index scores are used for analysis.

### Ordinary least squares regression analysis

To test the three structural hypotheses linking the three layers of the analytical framework, associations among the three indices were estimated using OLS regressions, applying the equation $Y_{i}=\beta_{0}+\beta_{1}X_{1i}+\beta_{2}X_{2i}+\varepsilon_{i}.$

The operational OLS regression equation is stipulated as follows:

$${ACRI}_{i}=\beta_{0}+\beta_{1}*{RPI}_{i}+\beta_{2}*MRCI_{i}+\varepsilon_{i},$$ (2)

where ACRI is modelled as a function of RPI and MRCI, *β* parameters are estimated coefficients, and ^*ε*^ *i* is the error term (Equation 2). Because the study used cross-sectional observational data, the OLS results strictly represent the patterns of associations and not the causal effects. Robust standard errors were used to reduce sensitivity to heteroskedasticity.

### Unsupervised clustering and selection of the final cluster solution

To identify latent readiness maturity regimes, three unsupervised clustering algorithms–using *K*-means, agglomerative hierarchical clustering, and Gaussian mixture modelling (GMM)—were employed on the standardised indices space. The cluster profiles were interpreted extensively by estimating the relative contributions of RPI, MRCI, and ACRI.

The objective function is presented below:

$$\min\;C\;\sum_{k=1}^{K}\sum_{i\in C\_k}{\left\|x\_i-\left.\mu \_k\right\|\right.}^{2},$$ (3)

$$p\left(x\_i\right)=\sum_{k=1}^{K}\pi_{k}\;N\left(x\_i\;|\;\mu_{k},\Sigma_{k}\right),$$

$$s\left(i\right)=\frac{b\left(i\right)-a\left(i\right)}{\max\left\{a\left(i\right),b\left(i\right)\right\}},$$ (4)

where *x*_*i*_ is the feature vector space and *µ*_*k*_ represents cluster-specific centroids (Equation 3). Silhouette scores are used to assess clustering quality and regime separability, where *a*(*i*) is the average intra-cluster distance and *b*(*i*) is the lowest average inter-cluster distance (Equation 4). The cluster solutions were compared; a four-cluster configuration was adopted for established group comparison analysis and a five-cluster solution was adopted for recoverability analysis after cluster quality assessment using silhouette-based comparison. *K*-means clustering grouped countries into maturity regimes by minimising within-cluster variance in the multidimensional readiness space defined by RPI, MRCI, and ACRI. Agglomerative (hierarchical) clustering clustered the countries in a bottom-up manner, starting with each country as a cluster and then, based on a linkage criterion, merging the two closest clusters while minimising within-cluster variance. GMM estimates cluster memberships using maximum-likelihood, applying the expectation–maximisation algorithm, where data are generated from a mix of Gaussian distributions. It allows more flexibility in terms of overlapping cluster boundaries, shapes, and variances to build flexible heterogeneous structures.

### Robustness through external validity

The robustness and interpretability of the readiness clusters were evaluated by applying external validation and post-clustering recoverability analysis. External validation compared cluster formations with established country groupings to assess substantial plausibility. Cramér’s *V* (chi-square association test) was applied to test how strongly the derived clusters align with the World Bank country groupings and WHO regions. The Kruskal–Wallis test was also applied to evaluate whether the distribution of index scores varied across the World Bank and WHO groups without normality assumption.

### Robustness analysis for missing data handling

To assess the sensitivity of the results, the full analytical pipeline was repeated across the different missing data scenarios mentioned above. Robustness was compared to assess whether country-level readiness heterogeneity remained stable in a broader sense under these different scenarios. The analyses were conducted on

1. PCA loading structure and index relationships in the OLS models,
2. Preferred clustering composition, and
3. Broad cluster composition patterns.

### Post-clustering recoverability analysis

Recoverability analysis was conducted using four supervised learning models—logistic regression, random forest, decision tree, and XGBoost—to predict cluster membership using different evaluation metrics, where a five-cluster solution is considered for analysis to maintain compatibility with WHO’s five-category digital-health maturity phases.

The classification function (Equation 5) applied for prediction is defined as

$$Y\_\text{pred}=f\left(\text{RPI},\text{MRCI},\text{ACRI}\right),$$ (5)

PY=k | X=e^β_k^T X∑_j e^β_j^T X,

$$ŷ=\frac{\sum_{t=1}^{T}f\_t\left(x\right)}{T},$$

$$F1=\frac{2\;\cdot \;Precision\;\cdot \;Recall}{Precision+Recall}.$$

Model performance was evaluated using 50-fold cross-validation and metrics including accuracy, precision, and recall macro; macro-averaged *F1* score; and area under the receiver operating characteristic curve (AUROC). Collectively, these steps offer a careful and comprehensive assessment of whether the latent readiness regimes are statistically identifiable, stable, and recoverable within the composed readiness space.

## Results

**FIGURE 3:**
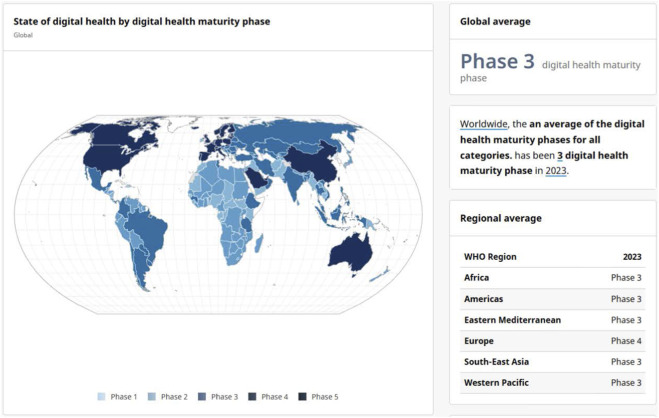
Global digital-health maturity status 2023. Source: World Helath Organization (2024), licensed under CC BY 4.0.

Global digital-health maturity status is presented in Figure 3. Globally, digital-health maturity reached phase 3—“defined/established.” Except for the EURO region (phase 4), all other WHO regions are at phase 3.

### Global analysis of multilayer robotic readiness of health systems

The latent structure of the three readiness indices derived from GDHM indicators is constructed using PCA under three data handling scenarios (Table 1). The perception/interoperability readiness dimension has shown a coherent loading profile, with the largest contributions arising from standards and interoperability, digital architecture, registry systems, secure identity systems, and scaling of population health-oriented digital systems. The governance/coordination dimension was dominated by indicators related to governance structure, strategic alignment, national planning, prioritisation of transformation, and funding commitments. The workforce–regulatory reliability dimension showed strong contributions from health worker training, digital maturity level among them, cross-border security, and AI and regulatory safeguards related to technology.

**TABLE 1:** PCA loading structure under different scenarios.

Scenario	No. of countries	Missing note	RPI PCA loading 1 explained	MRCI PCA loading 1 explained	ACRI PCA loading 1 explained
Baseline floor 1	169	Missing values replaced with 1 for core indicators	0.412	0.569	0.589
Complete case	33	Complete-case analysis using only observed core indicators	0.559	0.550	0.531
Observed only	169	Observed-only scenario with missing values retained until model-specific handling	0.415	0.390	0.489

Evidently, the structures of the three indices remained stable across the three scenarios; however, some variation in explained variance was observed. Under the baseline floor-coded scenario (*n* = 169), the first component explained 41.2% (RPI), 56.9% by MRCI, and 58.9% by ACRI of the variance. In the complete-case sample (*n* = 33), explanatory power of RPI increased (55.9%) and became comparable with MRCI (55.0%) but remained lower than ACRI (53.1%). Under the observed-only scenario (n = 169), variance explained was 41.5% (RPI), 39.0% (MRCI), and 48.9% (ACRI). Overall, despite the possibility of missing data handling-related sensitivity, the dimensional structure remains interpretable across scenarios.

Table 2 demonstrates the association between ACRI, RPI, and MRCI, measured using OLS. In Model 1, the RPI is significantly positively associated with ACRI (*β* = 0.738, *p* < 0.01) in the baseline scenario, which increases in complete case (*β* = 0.844, *p* < 0.01) and observed-only (*β* = 0.773, *p* < 0.01) scenarios. This signifies that irrespective of the scenarios, higher levels of DHM in the national digital architecture, information exchange system, network, and infrastructural readiness are strongly aligned with greater system reliability for implementation of autonomous computing capacity.

**TABLE 2:** Index relationships in the OLS models.

Scenario	No. of countries	Missing note	RPI PCA loading 1 explained	MRCI PCA loading 1 explained	ACRI PCA loading 1 explained	OLS1 RPI *β*	OLS2 RPI *β*	OLS2 MRCI *β*	OLS2_R-squared
Baseline floor 1	169	Missing values replaced with 1 for core indicators	0.412	0.569	0.589	0.738***	0.142***	0.838***	0.892
Complete case	33	Complete-case analysis using only observed core indicators	0.559	0.550	0.531	0.844***	0.308*	0.645***	0.841
Observed only	169	Observed-only scenario with missing values retained until model-specific handling	0.415	0.390	0.489	0.773***	0.343***	0.549***	0.713

Legend: *p < 0.1; **p < 0.05; ***p < 0.01.

In Model 2, after introducing MRCI, the magnitude of association with perception readiness falls but remains significantly positive (*β* = 0.343, *p* < 0.01). Maturity in coordination readiness demonstrates a stronger and significant association with ACRI (*β* = 0.838, *p* < 0.01). Model fit increases markedly in Model 2 after inclusion of coordination readiness (*R* ^2^ = 0.892, *p* < 0.001; *N* = 169) with the highest explainability achieved in baseline scenario. Therefore, the results reveal that governance mechanisms and strategic investments are positively associated with building an enabling environment for mature intelligent robotics implementation, accounting for a substantial proportion of variance in system regulatory reliability across scenarios.

**FIGURE 4:**
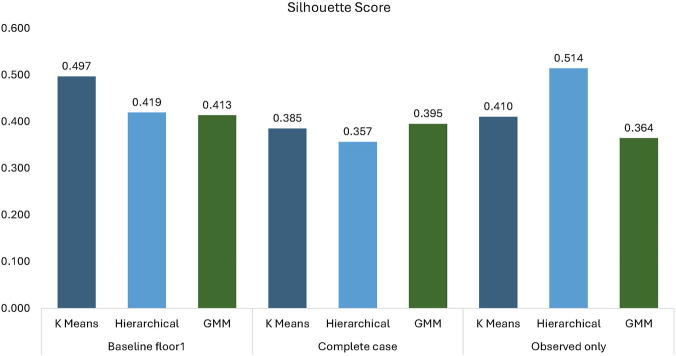
Cluster quality assessment applying Silhouette scores.

Clear cluster distinctiveness, assessed using the silhouette coefficient, is visible across missing data scenarios (Figure 4). Under the baseline floor-coded scenario, *K*-means achieved the highest distinctiveness (0.497), followed by hierarchical clustering (0.419) and GMM (0.413). Clustering performance in the complete-case scenario was slightly lower (*K*-means: 0.385; hierarchical: 0.357; GMM: 0.395). In contrast, the observed-only scenario showed better distinctiveness for hierarchical clustering (0.514), outperforming *K*-means (0.410) and GMM (0.364). Therefore, the results indicate method sensitivity to missing data handling. Overall, the baseline scenario is indicative of structurally distinctive but well-predicted national cases.

### Robustness through external validity

External validation shows significant alignment of *K*-means and GMM readiness regimes with the five established World Bank country groups and seven WHO regions (Table 3). The predominant concentration of high-income countries in the “no reported readiness” group (*n* = 45) and of low-income countries in the “high readiness” cluster (*n* = 12), indicate non-linear patterns in readiness distribution. The higher polarisation of Europe and Central Asia in “no reported readiness” (n = 43) is concordant with the skewed dominance of high-income countries in the same latent regime. Transitional disparity is reflected in more dispersed presence of upper- and lower-middle-income countries across clusters, which is in line with the balanced distributions of East Asia and the Pacific, Middle East, and Sub-Saharan African regions across readiness levels.

**TABLE 3:** Cross-tabulation of readiness clusters by World Bank income groups and WHO regions.

Country specification by the World Bank income groups	No reported readiness	Low readiness	Moderate readiness	High readiness
High income	44.1	14.3	26.3	5.9
Low income	5.9	14.3	10.5	35.3
Lower middle income	16.7	57.1	26.3	44.1
Not classified	1.0	0.0	5.3	0.0
Upper middle income	32.4	14.3	31.6	14.7
Country specification by the WHO regions	No reported readiness	Low readiness	Moderate readiness	High readiness
East Asia and Pacific	11.8	7.1	26.3	14.7
Europe and Central Asia	42.2	0.0	10.5	2.9
Latin America and Caribbean	21.6	14.3	15.8	5.9
Middle East, North Africa, Afghanistan, and Pakistan	11.8	7.1	15.8	11.8
North America	2.0	0.0	0.0	0.0
South Asia	2.0	14.3	5.3	2.9
Sub-Saharan Africa	8.8	57.1	26.3	61.8

The associations between derived latent regimes and established country groupings are statistically significant according to the chi-square test of independence, with moderate strength for *K*-means (Cramér’s *V* = 0.389) and weaker alignment with GMM (*V* = 0.277) (Table 4). Comparatively, geographical coherence in clustering is stronger than alignment with economic classifications—as evident from Cramér’s *V* with WHO regions (*K*-means *V* = 0.432; GMM *V* = 0.322) (Table 4). Kruskal–Wallis tests further indicate that RPI significantly differentiates groups (*H* = 26.5, *p* < 0.001; *H* = 16.5, *p* = 0.011), whereas MRCI and ACRI show significance mainly across regional groupings (Table 5).

**TABLE 4:** External validation of readiness clusters against World Bank income groups and WHO regional classifications (chi-square and Cramér’s *V* results).

Clustering method	Grouping	No. of countries	No. of groups	No. of clusters	Chi-square	*p-*value	*df*	Cramér’s *V* test
*K*-means	World Bank classification	169	5	4	87.3493	0.0000	12	0.389
	WHO regions	169	7	4	110.8563	0.0000	18	0.432
GMM	World Bank classification	169	5	4	50.3866	0.0000	12	0.277
	WHO regions	169	7	4	69.7385	0.0000	18	0.322

**TABLE 5:** Non-parametric comparison of readiness indices across country groupings (Kruskal–Wallis test results).

Index	No. of countries	No. of groups	Kruskal statistics	*p-*value
RPI_100	169	5	26.5	0.0000
MRCI_100	169	5	7.5	0.1136
ACRI_100	169	5	5.4	0.2473
RPI_100	169	7	16.5	0.0114
MRCI_100	169	7	23.6	0.0006
ACRI_100	169	7	24.0	0.0005

The distribution of index contributions across *K*-means, GMM, and the three scenarios reflects consistent structural patterns with a few variations (Table 6). Under the baseline floor-coded scenario, *K*-means depicts a strongly polarised structure with respect to RPI share dominance in the “high readiness” (82.7%) and “no reported readiness” clusters (61.6%). Remarkably minimal contributions from MRCI (8.6%) and ACRI (8.7%) are evident under the *K*-means “high readiness” and GMM “no reported readiness” (MRCI [7.8%] and ACRI [7.3%]) clusters. In contrast, a more balanced structure is visible in other GMM clusters, except for RPI heaviness in the “no readiness” cluster (84.9%) compared with balanced distributions in other clusters (moderate: RPI 32.7%, MRCI 37.4%, ACRI 29.9%).

**TABLE 6:** Cluster composition across readiness regimes and missing data scenarios—four-cluster solutions.

*K*-means clustering	RPI share	MRCI share	ACRI share	No. of countries	Scenario	Method	GMM clustering
No reported readiness	61.6	25.8	12.5	62	Baseline floor 1	*K*-means	
Low readiness	32.7	36.8	30.5	17	Baseline floor 1	*K*-means	
Moderate readiness	30.8	38.1	31.1	34	Baseline floor 1	*K*-means	
High readiness	82.7	8.6	8.7	56	Baseline floor 1	*K*-means	
	84.9	7.8	7.3	102	Baseline floor 1	GMM	No reported readiness
	36.6	32.0	31.5	14	Baseline floor 1	GMM	Low readiness
	32.7	37.4	29.9	19	Baseline floor 1	GMM	Moderate readiness
	26.0	44.2	29.7	34	Baseline floor 1	GMM	High readiness
No reported readiness	32.2	34.4	33.4	3	Complete case	*K*-means	
Low readiness	35.4	35.6	29.1	13	Complete case	*K*-means	
Moderate readiness	32.4	38.4	29.1	10	Complete case	*K*-means	
High readiness	26.1	36.0	37.9	7	Complete case	*K*-means	
	34.9	37.8	27.3	6	Complete case	GMM	No reported readiness
	33.4	35.7	30.9	21	Complete case	GMM	Low readiness
	28.3	39.5	32.3	4	Complete case	GMM	Moderate readiness
	33.2	33.2	33.6	2	Complete case	GMM	High readiness
No reported readiness	31.3	40.8	27.9	77	Observed only	*K*-means	
Low readiness	32.1	37.0	30.9	14	Observed only	*K*-means	
Moderate readiness	37.8	35.9	26.3	55	Observed only	*K*-means	
High readiness	26.2	45.2	28.6	23	Observed only	*K*-means	
	29.8	39.7	30.5	50	Observed only	GMM	No reported readiness
	30.1	35.7	34.2	5	Observed only	GMM	Low readiness
	34.7	37.5	27.9	13	Observed only	GMM	Moderate readiness
	35.0	38.9	26.1	101	Observed only	GMM	High readiness

In the complete-case scenario (n = 33), both clustering methods produce more balanced index shares across clusters, with contributions mostly within a 30%–38% distribution range, indicating balanced dominance of all indices. For instance, ACRI prominence (37.9%) is evident in *K*-means “high readiness,” with relatively even distribution across GMM clusters (e.g., high: ∼33% across all indices).

Under the observed-only scenario, *K*-means shows MRCI prominence in “high readiness” (45.2%) and “no reported readiness” (40.8%), reflecting that governance and coordination components gain relevance as raw data are preserved. GMM yields polarised clusters, with the “high readiness” group (n = 101) having a relatively balanced contribution of RPI (35.0%) and MRCI (38.9%). Overall, *K*-means produces more skewed cluster compositions, whereas GMM yields comparatively balanced compositions across scenarios.

### Recoverability analysis

A five-cluster solution is adopted for the recoverability analysis based on two reasons—first, digital-health maturity consists of five maturity phases; thus, a five-cluster solution reflects policy relevance. Second, as per cluster quality analysis, *K*-means silhouette scores differ marginally between four-cluster (0.633) and five-cluster (0.617) solutions (Table 7).

**TABLE 7:** Cluster quality analysis.

Method	k	Silhouette
*K*-means	5	0.617
Hierarchical	5	0.583
GMM	5	0.567

Across the three clustering methods, the composition of readiness scores reflects clear variations in how the three indices contribute under the baseline scenario (Table 8). *K*-means yields a dominant “low readiness” cluster (*n* = 111) that contributed largely by ACRI (57.2%), whereas higher readiness clusters show stronger governance and coordination influence (50.2% in “very good readiness”). Hierarchical clustering shifts this pattern towards stronger workforce–regulatory influence in the large “low to moderate readiness” group (*n* = 104) with very high ACRI dominance (70.0%). In contrast, GMM identifies similar ACRI (72.0%) prominence on a very large ‘no data to least readiness’ cluster (*n* = 102), whereas balanced contributions from MRCI is visible in higher readiness groups.

**TABLE 8:** Cluster composition across readiness regimes baseline missing data scenario—five-cluster solutions.

Clustering method	Readiness regime	RPI share (%)	MRCI share (%)	ACRI share (%)	No. of countries in cluster
*K*-means	No data to least readiness	38.3	38.2	23.4	18
	Low readiness	26.1	16.7	57.2	111
	Low to moderate readiness	38.5	38.4	23.1	9
	Moderate to good readiness	31.7	34.9	33.3	6
	Very good readiness	34.2	50.2	15.6	25
Hierarchical	No data to least readiness	37.8	36.4	25.8	19
	Low readiness	39.4	46.7	13.9	29
	Low to moderate readiness	18.2	11.8	70.0	104
	Moderate to good readiness	35.4	33.4	31.2	4
	Very good readiness	31.5	46.8	21.6	13
GMM	No data to least readiness	17.1	10.9	72.0	102
	Low readiness	43.6	34.8	21.6	15
	Low to moderate readiness	39.0	41.3	19.7	5
	Moderate to good readiness	37.0	48.7	14.3	34
	Very good readiness	29.4	37.4	33.2	13

The findings (Table 9) reveal strong recoverability of the labelled clustering structures using supervised classification algorithms. For the resultant clusters under *K*-means, XGBoost achieved the highest performance (accuracy = 0.980 and F1 = 0.966), followed by random forest (accuracy = 0.961 and F1 = 0.861) and decision tree (accuracy = 0.961 and F1 = 0.910), designating non-linear ensemble methods over single learners in effectively capturing the underlying cluster boundaries. Logistic regressions reflected relatively lower recall (0.810) but preserved high AUROC (0.999), signifying decent probabilistic separation despite decreased sensitivity. For GMM-derived clusters, logistic regressions and decision tree achieved the highest accuracy (0.980) and higher *F1* scores (0.986 and 0.971, respectively), signifying better linear separability of these clusters. XGBoost and random forest also performed well with high accuracy (0.961 and 0.922, respectively) and AUROC (0.999 and 0.998, respectively), substantiating robustness across single and ensemble learners. Overall, the consistency (high AUROC) across all models suggests very good separability and firm recoverability of latent readiness regimes. That said, to rule out the possibility of overfitting, 50-fold cross-validation was run, and results are presented in Table 10.

**TABLE 9:** Post-clustering classification performance for recoverability of latent readiness regimes.

Target	Model	Accuracy	Precision macro	Recall macro	F1 macro	AUROC macro OvR	Train set—no. of observations	Test set—no. of observations
*K*-means	Logistic regression	0.922	0.910	0.810	0.837	0.999	118	51
	Random forest	0.961	0.920	0.860	0.861	1.000	118	51
	XGBoost	0.980	0.978	0.960	0.966	1.000	118	51
	Decision tree	0.961	0.928	0.920	0.910	0.956	118	51
GMM	Logistic regression	0.980	0.994	0.980	0.986	0.999	118	51
	Random forest	0.922	0.920	0.860	0.872	0.998	118	51
	XGBoost	0.961	0.974	0.930	0.948	0.999	118	51
	Decision tree	0.980	0.967	0.980	0.971	0.988	118	51

**TABLE 10:** Cross-validated performance and generalisation assessment of post-clustering classification models (50-fold evaluation).

Model	Folds	Train accuracy means	Train accuracy SD	Test accuracy means	Test accuracy SD	Train F1 macro mean	Train F1 macro-SD	Test F1 macro mean	Test F1 macro-SD	Train AUC mean
Decision tree (*K*-means)	50	0.809	0.048	0.794	0.055	0.472	0.096	0.436	0.107	0.938
Logistic regression (*K*-means)	50	0.965	0.011	0.927	0.045	0.945	0.019	0.818	0.141	0.997
Random forest (*K*-means)	50	0.973	0.011	0.910	0.043	0.922	0.079	0.782	0.128	0.998
XGBoost (*K*-means)	50	0.985	0.010	0.907	0.040	0.849	0.106	0.747	0.128	0.999
Decision tree (GMM)	50	0.819	0.093	0.792	0.102	0.518	0.115	0.518	0.168	0.945
Logistic regression (GMM)	50	0.944	0.023	0.892	0.050	0.936	0.040	0.758	0.130	0.998
Random forest (GMM)	50	0.974	0.015	0.899	0.045	0.924	0.058	0.730	0.139	0.999
XGBoost (GMM)	50	0.981	0.012	0.912	0.047	0.852	0.115	0.814	0.119	0.999

Train AUC SD	Test AUC mean	Test AUC SD	Gap accuracy means	Gap accuracy SD	Gap F1 mean	Gap F1 SD	Gap AUC mean	Gap AUC SD	Silhouette train mean	Silhouette train SD
0.035	0.907	0.043	0.015	0.037	0.036	0.065	0.031	0.038	0.622	0.015
0.001	0.991	0.007	0.038	0.048	0.127	0.141	0.006	0.008	0.622	0.015
0.001	0.989	0.007	0.063	0.042	0.140	0.154	0.010	0.007	0.622	0.015
0.001	0.984	0.012	0.078	0.041	0.102	0.168	0.016	0.012	0.622	0.015
0.025	0.932	0.055	0.026	0.053	0.000	0.106	0.025	0.030	0.523	0.072
0.002	0.988	0.008	0.052	0.042	0.178	0.126	0.009	0.008	0.523	0.072
0.001	0.989	0.010	0.075	0.044	0.193	0.141	0.010	0.010	0.523	0.072
0.001	0.986	0.020	0.069	0.051	0.038	0.178	0.014	0.020	0.523	0.072

Across 50-fold cross-validation (Table 10), all models reveal strength and steadiness in performance, reflected in high test set accuracy (∼0.79–0.93) and almost consistently high AUROC (≥0.98). The logistic regression model depicts well-balanced generalisability (test accuracy up to 0.927; lowest AUC gap ≈ 0.006), whereas random forest and XGBoost achieve higher performance in the training set (train accuracy of up to 0.985) but marginally reduced generalisability (accuracy gap of up to 0.078). The variability in *F1* scores, particularly for XGBoost and random forest, denotes sensitivity to class imbalance. Silhouette scores stay consistent (0.622 vs. 0.523), signifying stability in cluster structure independent of the classification model.

**FIGURE 5:**
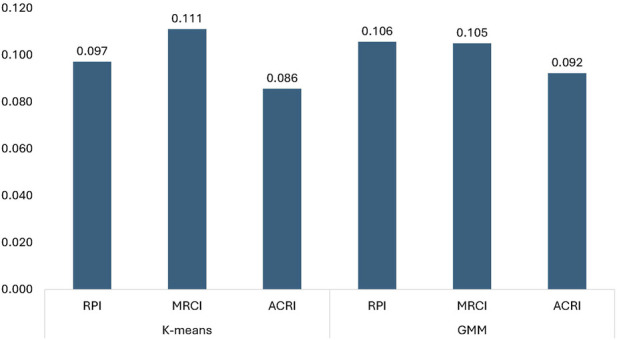
SHAP (SHapley Additive exPlanations)-based feature importance of readiness indices across clustering frameworks (reference model: random forest).

SHAP analysis (Figure 5) identified MRCI (0.111) as the strongest influencing factor of maturity regime classification under *K*-means. However, RPI is the most influential predictor of cluster classification under GMM (0.106), followed by MRCI and ACRI. Therefore, SHAP analysis demonstrates how feature importance varies across clustering methods, revealing that underlying clustering structure influences feature dominance in classifying country-level readiness regimes. In a nutshell, SHAP analysis reinforces the layered system architecture, where infrastructure, interoperability, coordinated leadership capacity and regulatory compliance, digital literacy, and workforce maturity influence cluster membership.

## Discussion

This study expands a systems-level insight into healthcare robotics readiness by empirically assessing digital-health maturity to determine structural readiness across 169 countries applying GDHM 2023 data. Three latent readiness dimensions—RPI, MRCI, and ACRI—were constructed and validated within a layered, theory-based analytical framework applying dimensionality reduction techniques (PCA), conventional OLS, and machine learning modelling. The findings highlight that the interactions of interoperable infrastructure, governance coordination, and workforce–regulatory capacity constitute the necessary condition for the system’s robotics readiness as a foundation for further technological sophistication.

The PCA results reveal that the readiness indices are statistically stable and explainable across three missing data handling scenarios, where RPI portrays initial interoperability and digital architecture as a prelude to MRCI, which indicates governance and coordination readiness, followed by ACRI, which reflects workforce and regulatory reliability. The requirement of infrastructural and interoperability readiness is consistent with prior evidence emphasising scalable digital architectures, identity management systems, and secure ethical data exchange as preconditions for advanced health-system capacities (Stoumpos et al., 2023; Erku et al., 2023). Furthermore, the emphasis on structural readiness aligns with robotics research suggesting that a specific standardised enabling environment, such as building structure, layout, and operational environment, is rudimentary for intelligent robotic systems. For example, the effectiveness of robotic sensing and perception depends on structured systemic conditions, and the achievement of higher maturity regimes augments accurate perception and decision-making (Huang et al., 2023; Hung et al., 2021). For instance, scalability is significantly constrained at early phases of digital maturity in the form of infrastructural instabilities—such as irregular electricity supply, limited access to efficient fault-tolerant hardware, or reliable maintainable software architecture, as mentioned by Alotaibi et al. (2025)—which supports the observed structural role of RPI.

The results indicate that perception readiness is one component of the necessary conditions to be in place towards robotics integration. The second element is governance and coordination readiness (MRCI), which emerges as an essential conjoining dimension, especially in higher maturity regimes. This aligns with previous research underscoring the role of strategic positioning, regulatory frameworks, and institutional coordination in enabling complex technology adoption and implementation, including human–robot collaboration (Bober et al., 2024; Sifakis, 2019).

Governance mechanisms, including alignment of policy and budget line priorities, have been shown to influence the successful adoption of intelligent systems such as cobots as artificially intelligent delivery transporters in critical care settings (Birkhoff et al., 2024). Moreover, health workforce preparedness and the digital literacy of healthcare professionals remain prerequisites for the system’s operational efficacy (Alotaibi et al., 2025). These findings collectively support a layered systemic foundation, rather than a purely mechanistic perspective of algorithmic or sensory advancements alone, as a precondition or necessary condition (Shin et al., 2025). Our findings empirically reinforce this theoretical framework on a global scale.

Therefore, the operationalisation of workforce–regulatory reliability (ACRI) further strengthens this interpretation, consistent with the unified theory of acceptance and use of technology (UTAUT). In alignment with UTAUT fundamentals, workforce competency, regulatory safeguards, and infrastructure reliability form the base for sustained adoption and use of advanced computational technologies (Alotaibi et al., 2025; Huang et al., 2023). The observed association between MRCI and ACRI reinforces the argument that coordinated governance positively conditions regulatory compliance, trust, and operational reliability. This is specifically valid in healthcare robotics, where verification, validation, and oversight mechanisms are crucial to ensure patients’ safety through effective implementation emphasising patient and provider confidence-building under uncertainty (Denecke and Baudoin, 2022). Thus, mature, trustworthy systems are preconditions for successful robotics applications spanning into areas such as older adult care, telemedicine of homecare support, and critical care.

Clustering analysis reveals moderate distinctiveness in readiness regimes, exhibiting structural heterogeneity across countries. The distinct separation of these latent regimes is consistent with theories of phased digital transformation and diffusion of innovation that emphasise non-linear stage progression and threshold effects (Stoumpos et al., 2023). Although *K*-means yields more differentiated groups and GMM produces smoother probabilistic clusters, both methods draw attention to the significance of infrastructure, governance, and coordination in transitioning to advanced readiness phases. External validation further demonstrates the relevance of the derived clusters, depicting moderate alignment with World Bank country groupings and stronger associations with WHO regions, designating the importance of regional and systemic factors in shaping readiness rather than economic growth alone.

The high predictive performance of supervised models (AUROC ≈ = 1.00), complemented by 50-fold cross-validation, confirms the structural stability and recoverability of the distinct readiness regimes. SHAP analysis describes the methodological and structural context-dependency of feature importance: MRCI depicts a stronger association under *K*-means clustering, whereas RPI dominates under GMM, with ACRI showing consistent but lesser contributions. This reinforces the need for algorithmic modifications (e.g., constrained or stratified *K*-means) to group regions and economic classifications with similar structural contexts to minimise heterogeneity in interactions among readiness dimensions.

Overall, the findings support a layered and non-linear model of robotics readiness, where interoperable infrastructure serves as the foundation, governance coordination enables integration, and workforce–regulatory capacity ensures reliable operation. Although the mechanistic view supports technological determinism as a sufficient condition for intelligent robotics deployment (Albekairi et al., 2025), the perspective shifts to system-centric readiness, emphasising institutional and structural reliability readiness as a sufficient enabling condition for sustainable robotics adoption in healthcare.

### Structural layering and cluster heterogeneity: maturity regimes in global robotics ecosystems

The discrete phases of maturity regimes created using *K*-means and GMM clustering models align with digital transformation stage theories and the diffusion of innovations that theorise phased non-linearity in technological adoption (Stoumpos et al., 2023). The layered architecture also corresponds to a multi-agent system hierarchy framework, where perception, coordination, and systemic autonomy contribute to progressive robotic readiness achievements. Though digital-health maturity is a phenomenon with a continuous curve of progression, regime-based structural clustering identified the significance of latent thresholds and systemic tipping points in robotics readiness transitions.

**FIGURE 6:**
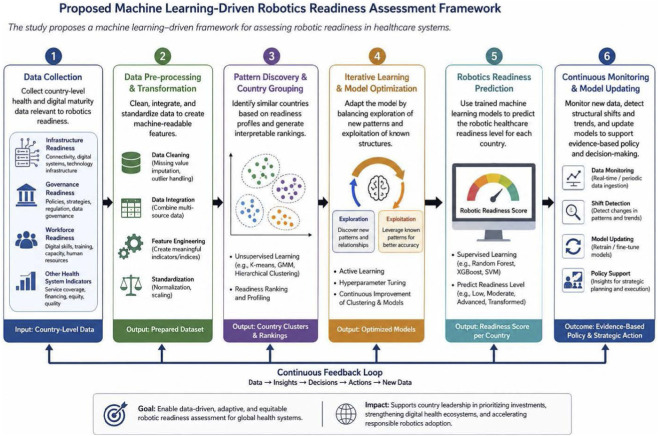
Proposed machine learning-driven robotics readiness assessment framework. Source: created by the authors using GPT 5.0.

The study proposes a machine learning-driven framework for assessing robotic readiness in healthcare systems (Figure 6).

1. The system begins by collecting country-level data on health systems and digital maturity, including infrastructure, governance, and workforce readiness indicators relevant to robotics adoption.
2. The collected data are then preprocessed and transformed into standardised, machine-readable formats to enable meaningful comparison and analysis.
3. The system subsequently identifies patterns in the data by grouping countries with similar readiness profiles and generating interpretable rankings.
4. Through iterative learning, the model adapts by balancing exploration of new patterns and exploitation of known structures, improving clustering and prediction over time.
5. Using trained machine learning models, the system predicts each country’s level of robotic healthcare readiness with high accuracy.
6. Finally, the system continuously monitors incoming data, detects structural shifts and trends, and updates model outputs to support evidence-based policymaking and strategic planning.

## Conclusion

This study tests and empirically validates a layered analytical framework for examining healthcare robotics readiness by applying global digital-health maturity data. The findings uncover a hierarchical structure in which interoperability and digital infrastructure (RPI) form the foundational layer, governance and coordination (MRCI) operates as the underlying integrative mechanism, and workforce–regulatory reliability (ACRI) enables ultimate autonomous system functionality. Regression and clustering analyses confirm the distinctiveness of and associations between these readiness dimensions, with governance readiness reflecting the strongest direct association with autonomous reliability.

The detection of distinct readiness regimes, supported by consistent recoverability (AUROC ∼ 0.99), and the alignment with established country group classifications highlights the analytical robustness. Notably, the findings emphasise that structural readiness is the precondition for successful adoption of technological advancements such as robotics adoption. Despite emerging advancements, most of the lower-middle-income countries remain in early-to-mid maturity stages, indicating substantial gaps in system-level preparedness or indeterminacy due to a lack of access to digital-health maturity status, especially driven by regulatory restrictions. Addressing these gaps requires globally coordinated governance exploring standard ethical interoperability mechanisms, investments in infrastructure, and workforce capacity to enable equitable and scalable integration of robotics in healthcare systems.

## Limitations

This study has several limitations. First, the analysis relies on GDHM data, which is self-declared administrative data submitted to WHO individually by WHO member states. The possibility of reporting bias cannot be eliminated, and variability in measurement accuracy across countries cannot be fully ignored. Second, the study was unable to capture any temporal transition in readiness or causal inference due to the cross-sectional nature of the dataset. Third, the readiness indices are constructed based on system-level structural indicators of digital-health maturity and cannot integrate robotics-specific implementation metrics due to data unavailability or inaccessibility. As a result, the three latent indices should be understood as proxy readiness dimensions relevant to future robotics integration, not as direct measures of robotics adoption, robotic service intensity, or clinical robotic performance. This is a conceptual limitation of the data source and should temper any strong claims about realised robotics deployment. Second, the analysis is cross-sectional. The OLS models identify associations among the proxy readiness dimensions, but they do not establish temporal ordering or causality. It is therefore not possible to conclude from the present data that stronger infrastructure or governance causes stronger workforce–regulatory readiness or that any of these dimensions directly cause future robotics adoption. Fourth, the treatment of missing or non-reported maturity information remains an important source of uncertainty. Although the study addresses this concern through robustness tests and sensitivity analyses across multiple scenarios, countries with no reported data still present a substantial challenge for comparative analysis. The robustness test results reduce the risk that the main findings are entirely driven by one arbitrary coding rule, but they do not remove all uncertainty surrounding countries with sparse or absent reporting. Borderline cases and intermediate cluster assignments may still be sensitive to how non-reported values are handled.

In addition, national-, regional-, and global-level geopolitical, demographic, and socioeconomic conditions; health financing structures and spending realities; and private sector engagements are not pooled for modelling, potentially limiting the ability to obtain multi-level non-linearities. Finally, user-level attributes such as patients’ knowledge, attitude and trust, belief and acceptance of robotics in healthcare were not assessed. These factors deserve attention in future research to ensure successful real-world implementation.

### Future research directions

Future research should focus on country and region-specific longitudinal data collection and analysis to capture temporal transitions between readiness regimes and identify structural tipping points over time. Periodic data collection from sample countries under each cluster would facilitate estimation of transition probabilities and forecasting of readiness trajectories. Integrating facility-level actual robotics deployment data with robotics adoption maturity data, degree of patient acceptance, and macro- and micro-level financing and governance structures would offer a more granular assessment of readiness dynamics.

Normative approaches should be adopted with implementation inclusive of trustworthy AI, ethical governance, and tech-equity frameworks to address equity dimensions, ensuring access to marginalised populations. Additionally, readiness indices should be redrawn, including real-world robotics deployment phases to strengthen the empirical validity of the framework. Therefore, future research on GDHM should extend to direct robotics implementation indicators to better connect structural digital-health maturity with realised robotics deployment outcomes. This is expected to inform the design of cluster-specific policy strategies to guide targeted interventions for effective progress towards different stages of robotics readiness while minimising the risk of widening digital and technological divides.

## Data Availability

The original contributions presented in the study are included in the article/Supplementary Material, further inquiries can be directed to the corresponding author.
