# Supplementary material for: Structural predictors and latent maturity regimes of robotic readiness in global health systems: evidence from machine learning-based latent clustering and class prediction
Source: Front Robot AI. 2026 Jul 16;13:1835473. doi: 10.3389/frobt.2026.1835473 (PMC13419970; doi:10.3389/frobt.2026.1835473)
Supplement: Supplementary file 1 [file Supplementaryfile1.docx]

**Table1: Variables applied to construct RPI**

| **Variable** | **Pillar** | **Phases of maturity** | **Description** |
| --- | --- | --- | --- |

|  | **Standards & Interoperability** |  |  |
| --- | --- | --- | --- |
| Variable 20 | Indicator 15 -National digital health architecture and/or health information exchange | Phase 1 | There is no national digital health (eHealth) architectural framework and/or health information exchange (HIE) established. |
|  |  | Phase 2 | A national digital health architecture and/or health information exchange (HIE) has been proposed, but not approved including semantic, syntactic, and organizational layers. |
|  |  | Phase 3 | The national digital health architecture and/or health information exchange (HIE) is operable and provides core functions, such as authentication, translation, storage and warehousing function, guide to what data is available and how to access it, and data interpretation. |
|  |  | Phase 4 | The government leads, manages, and enforces implementation of the national digital health architecture and/or the health information exchange (HIE), which are fully implemented following industry standards. |
|  |  | Phase 5 | The national digital health architecture and/or health information exchange (HIE) provides core data exchange functions and is periodically reviewed and updated to meet the needs of the changing digital health architecture. There is continuous learning, innovation, and quality control. Data is actively used for national health strategic planning and budgeting. |
| Variable 21 | Indicator 16 -Health information standards | Phase 1 | There are no digital health / health information standards for data exchange, transmission, messaging, security, privacy, and hardware. |
|  |  | Phase 2 | There are some digital health / health information standards for data exchange, transmission, messaging, security, privacy, and hardware that have been adopted and/or are used. |
|  |  | Phase 3 | Digital health / health information standards for data exchange, transmission, messaging, security, privacy, and hardware have been published and disseminated in the country under the government’s leadership. |
|  |  | Phase 4 | Digital health / health information industry-based technical standards for data exchange, transmission, messaging, security, privacy, and hardware are in use in the majority of applications and systems to ensure the availability (and use) of high-quality data. Conformance testing is routinely carried out to certify implementers. |
|  |  | Phase 5 | Data standards are routinely updated and data is actively used for monitoring and evaluating the health system and for national health strategic planning and budgeting. |
|  | **Infrastructure** |  |  |
| Variable 22 | Indicator 17 -Network readiness [network readiness index score (https://networkreadinessindex.org )] | Phase 1 | 0 |
|  |  | Phase 2 | < 25 |
|  |  | Phase 3 | 26 - 50 |
|  |  | Phase 4 | 51 - 75 |
|  |  | Phase 5 | > 75 |
| Variable 23 | Indicator 18 -Planning and support for ongoing digital health infrastructure maintenance | Phase 1 | There is no articulated plan for supporting digital health infrastructure (including equipment- computers/ tablets/ phones, supplies, software, devices, etc.) provision and maintenance. |
|  |  | Phase 2 | A plan for supporting digital health infrastructure (including equipment- computers/ tablets/ phones, supplies, software, devices, etc.) provision and maintenance has been developed, but not implemented. |
|  |  | Phase 3 | A plan for supporting digital health infrastructure (including equipment- computers/ tablets/ phones, supplies, software, devices, etc.) provision and maintenance has been implemented partially, but not consistently with estimated 0-25% of necessary digital health infrastructure needed in public healthcare service sector available and in use. |
|  |  | Phase 4 | A plan for supporting digital health infrastructure (including equipment- computers/ tablets/ phones, supplies, software, devices, etc.) provision and maintenance has been implemented partially and consistently with estimated 25-50% of necessary digital health infrastructure needed in public healthcare service sector available and in use. |
|  |  | Phase 5 | Digital health infrastructure (including equipment- computers/ tablets/ phones, supplies, software, devices, etc.) is available, in use, and regularly maintained and upgraded in >75% of public healthcare service sector. Strategies are being implemented to close outstanding gaps in access/coverage and reach the whole population. |
|  | **Services & Applications** |  |  |
| Variable 24 | Indicator 19 -Nationally scaled digital health systems | Phase 1 | National priority areas are not supported by digital health at any scale. |
|  |  | Phase 2 | Few national priority areas are supported by digital health, and implementation initiated (< 25% priority areas). |
|  |  | Phase 3 | Some national priority areas supported by scaled digital health systems (25-50% of priority areas). |
|  |  | Phase 4 | The majority, but not all national priority areas (50-75% of priority areas) supported by scaled digital health systems. |
|  |  | Phase 5 | All nationally prioritized areas supported by national-scale digital health systems (>75%) with monitoring and evaluation systems and results. |
| Variable 25 | Indicator 20 -Digital identity management of service providers, administrators, and facilities for digital health, including location data for GIS mapping | Phase 1 | Health system registries of uniquely identifiable providers, administrators, and public facilities (and private if applicable) are not available, accessible and current. |
|  |  | Phase 2 | Health system registries of uniquely identifiable providers, administrators, and public facilities (and private if applicable) are being developed but are not available for use. |
|  |  | Phase 3 | Health system registries of uniquely identifiable providers, administrators, and public facilities (and private if applicable) are available for use, but incomplete, partially available, used sporadically, and irregularly maintained. |
|  |  | Phase 4 | Health system registries of uniquely identifiable providers, administrators, and public facilities (and private if applicable) are available, used, and regularly updated and maintained. The data is geo-tagged to enable GIS mapping. |
|  |  | Phase 5 | Geotagged Health system registries of uniquely identifiable providers, administrators, and public facilities (and private if applicable) are available for all transactions, systematically used, and regularly updated, maintained, monitored, and evaluated. |
| Variable 26 | Indicator 21 -Digital identity management of individuals for health - MPI registry | Phase 1 | No secure registry or master patient index exists. |
|  |  | Phase 2 | A secure registry exists, but is incomplete / partially available, used, and irregularly maintained. |
|  |  | Phase 3 | A secure registry exists, is available and in active use and includes <25% of the relevant population. |
|  |  | Phase 4 | A secure registry exists, is available and in active use and includes 25-50% of the relevant population. |
|  |  | Phase 5 | A secure registry exists, is available and in active use and includes >75% of the relevant population. The data is available, used, and curated. Strategies are being implemented to include missing data and ensure fully representative datasets are available. |
| Variable 27 | Indicator 21a -Digital identity management of individuals for health - secure MPI register | Phase 1 | No secure master patient index exists. |
|  |  | Phase 2 | A master patient index exists, but is incomplete / partially available, used, and irregularly maintained. |
|  |  | Phase 3 | A master patient index exists, is available and in active use and includes <25% of the relevant population. |
|  |  | Phase 4 | A master patient index exists, is available and in active use and includes 25-50% of the relevant population. |
|  |  | Phase 5 | A master patient index exists, is available and in active use and includes >75% of the relevant population. The data is available, used, and curated. Strategies are being implemented to include 100% of the population. |
| Variable 28 | Indicator 21b -Digital identity management of individuals for health - Birth registry | Phase 1 | No secure birth registry exists. |
|  |  | Phase 2 | A secure birth registry exists, but is incomplete / partially available, used, and irregularly maintained. |
|  |  | Phase 3 | A secure birth registry exists, is available and in active use and includes <25% of the relevant population. |
|  |  | Phase 4 | A secure birth registry exists, is available and in active use and includes 25-50% of the relevant population. |
|  |  | Phase 5 | A secure birth registry exists, is available and in active use and includes >75% of the relevant population. The data is available, used, and curated. Strategies are being implemented to include 100% of the population. |
| Variable 29 | Indicator 21c -Digital identity management of individuals for health - death registry | Phase 1 | No secure death registry exists. |
|  |  | Phase 2 | A secure death registry exists, but is incomplete / partially available, used, and irregularly maintained. |
|  |  | Phase 3 | A secure death registry exists, is available and in active use and includes <25% of the relevant population. |
|  |  | Phase 4 | A secure death registry exists, is available and in active use and includes 25-50% of the relevant population. |
|  |  | Phase 5 | A secure death registry exists, is available and in active use and includes >75% of the relevant population. The data is available, used, and curated. Strategies are being implemented to include 100% of the population. |
| Variable 30 | Indicator 22 -Secure Patient Feedback Systems | Phase 1 | No secure patient feedback system exists. |
|  |  | Phase 2 | A secure patient feedback system exists for some health services, but is incomplete/ partially available, used, and irregularly maintained. |
|  |  | Phase 3 | A secure feedback system exists, is available and in active use and includes data from <25% of the relevant health services and/or geographic location. It is available to some of the population. |
|  |  | Phase 4 | A secure feedback system exists, is available and in active use and includes data from 25-75% of the relevant health services and/or geographic location. It is available to most of the population. |
|  |  | Phase 5 | A secure feedback system exists, is available in accessible formats and in active use and includes data from >75% of the relevant health services and/or geographic location. It is available to 100% of the population. |
| Variable 31 | Indicator 23 -Population health management contribution of digital health | Phase 1 | No contribution from any digital health initiative to routine reporting or for data-based decision making for population health management. |
|  |  | Phase 2 | Digital systems used at district/state levels only contribute to country public health reporting and decision making for population health management. |
|  |  | Phase 3 | Digital systems are used at facility or community levels and contribute to the country's public health reporting and decision-making for population health management. |
|  |  | Phase 4 | Digital systems are used at facilities or communities and by individuals and contribute to the country's public health reporting and decision-making for population health management. |
|  |  | Phase 5 | Digital systems used at all levels, including by individuals, contribute to timely country public health reporting and decision making for population health management. |

**Table 2:** **Variables applied to construct MRCI**

| **Variable** | **Pillar** | **Phases of maturity** | **Description** |
| --- | --- | --- | --- |
|  | **Leadership & Governance** |  |  |
| Variable 1 | Indicator 1 - Digital health prioritized at the national level through dedicated bodies / mechanisms for governance | Phase 1 | No coordinating body exists and/or nascent governance structure for digital health is constituted on a case-by-case basis. |
|  |  | Phase 2 | Governance structure is formally constituted though not fully-functional or meeting regularly. |
|  |  | Phase 3 | Governance structure and any related working groups have a scope of work (SOW) and conduct regular meetings with stakeholder participation and/or consultation. |
|  |  | Phase 4 | Governance structure is fully-functional, government-led, consults with other ministries, and monitors implementation of digital health and data governance, including artificial intelligence, based on a work plan. |
|  |  | Phase 5 | The digital health and data governance structure is institutionalized, consults with other ministries, and monitors implementation of digital health. It is relatively protected from interference or organizational changes. It is nationally recognized as the lead for digital health. The governance structure and its technical working groups emphasize gender balance in membership. |
| Variable 2 | Indicator 2 - Digital Health prioritized at the national level through planning | Phase 1 | Digital health is not included in the national health strategy. It is being implemented in an ad hoc fashion in health programs. |
|  |  | Phase 2 | There is some discussion of inclusion of digital health in national health or other relevant national strategies or plans. Proposed language for inclusion of digital health in national health or relevant national strategies and/or plans has been made and is under review. |
|  |  | Phase 3 | Digital health is included in national health or relevant national strategies and/or plans. |
|  |  | Phase 4 | Digital health is being implemented as part of national health or other relevant national strategies and/or plans. |
|  |  | Phase 5 | Digital health is implemented and periodically evaluated and optimized in national health or other relevant national strategies and/or plans. |
| Variable 3 | Indicator 2a- Health is prioritized in national digital transformation and data governance policies | Phase 1 | National digital transformation and data governance policies do not include potential benefits and risks on public health or individual health outcomes. |
|  |  | Phase 2 | National digital transformation and data governance policies include potential benefits and risks on public health or individual health outcomes on an ad hoc basis. |
|  |  | Phase 3 | National digital transformation and data governance policies systematically includes potential benefits and risks on public health or individual health outcomes but has no strategy(s) for addressing them. |
|  |  | Phase 4 | National digital transformation and data governance policies systematically includes potential benefits and risks on public health or individual health outcomes and has some strategy(s) for addressing them that are not yet implemented. |
|  |  | Phase 5 | All relevant digital transformation and data governance policies fully consider potential benefits and risks for health systems, determinants of health and individual health outcomes (including for children, women and vulnerable groups) and have multisectoral strategies that are fully implemented. |
| Variable 4 | Indicator 3 -Readiness for emerging technologies adoption and governance | Phase 1 | There is no emerging technologies (e.g., AI, Wearables, Blockchain, IoT) plan in support of public health goals. |
|  |  | Phase 2 | A plan was developed for at least one emerging technology (e.g., AI, Wearables, Blockchain, IoT) to support public health goals, but it is not being implemented. |
|  |  | Phase 3 | A plan exists for at least one emerging technology (e.g., AI, Wearables, Blockchain, IoT) to support public health goals. Plan(s) identifies governance mechanisms required for emerging technologies. |
|  |  | Phase 4 | A plan for one or more emerging technology (e.g., AI, Wearables, Blockchain, IoT) to support public health is being implemented, is funded, and the results are being monitored. Governance mechanisms required for emerging technologies are in place. |
|  |  | Phase 5 | A plan for one or more emerging technology (e.g., AI, Wearables, Blockchain, IoT) in support of public health is being implemented, is funded, the results are being monitored, and the plan is kept updated. Governance mechanisms are in place and in use. |
| Variable 5 | Indicator 4 -Diversity, Equity, and human rights analysis, planning and monitoring included in national digital health strategies and plans | Phase 1 | Digital health strategies and programs are developed and implemented without consideration of equity and human rights implications. |
|  |  | Phase 2 | Digital health strategies and programs are developed and implemented with ad hoc consideration of equity and human rights implications. |
|  |  | Phase 3 | Digital health strategies and programs are developed and implemented with formal consideration of equity and human rights implications with no strategy(s) for addressing them. |
|  |  | Phase 4 | Digital health strategies and programs are developed and implemented with formal consideration of equity and human rights implications with ad hoc strategy(s) for addressing them. |
|  |  | Phase 5 | The country is implementing and evaluating the effects of digital health strategies and specific digital health solutions based on equity and human rights impact assessments. Documented strategies are in place to address gaps in access and outcomes for different population groups, including women, children, and marginalized groups. |
| Variable 6 | Indicator 4a -Gender considerations accounted for in digital health strategies and digital health governance | Phase 1 | Digital health strategies and interventions are developed and implemented without consideration of gender norms, roles, and relations. |
|  |  | Phase 2 | Digital health strategies and interventions are developed and implemented with ad hoc and non-systematic consideration of gender norms, roles, and relations. |
|  |  | Phase 3 | Digital health strategies and programs are developed and implemented with systematic consideration of gender norms, roles, and relations without the policies or structures for accountability (gender-sensitive). |
|  |  | Phase 4 | Digital health strategies and programs are developed and implemented with systematic consideration of gender norms, roles, and relations with specific strategies targeting governance bodies, health workforce, and clients with mechanisms for accountability (gender-specific). |
|  |  | Phase 5 | Digital health strategies, policies, and interventions address gender inequality and foster progressive change in gender dynamics based on routine gender analyses and impact assessments (gender transformative). |
|  | **Strategy & Investment** |  |  |
| Variable 7 | Indicator 5 -National eHealth/ Digital Health Strategy or Framework | Phase 1 | There is no digital health strategy or framework. Draft digital health strategy or framework developed, but not officially reviewed. |
|  |  | Phase 2 | National digital health strategy or framework approved. |
|  |  | Phase 3 | National digital health costed plan developed and approved. |
|  |  | Phase 4 | National digital health strategy and costed plan partially implemented with resources to ensure full implementation. |
|  |  | Phase 5 | National digital health strategy and costed plan fully implemented with planning underway for the next 3-5 year cycle. |
| Variable 8 | Indicator 5a -National digital strategy alignment with Universal Health Coverage (UHC) Core Components | Phase 1 | Digital health strategy does not exist or exists and is not aligned to UHC and does not address any of the core UHC components of coverage, access, uptake, quality, and equity. |
|  |  | Phase 2 | Digital health strategy exists and is partly aligned to UHC, addressing only one core UHC component of coverage, access, uptake, quality, and equity. |
|  |  | Phase 3 | Digital health strategy exists and is partly aligned to UHC, addressing two or more core UHC components of coverage, access, uptake, quality, and equity. |
|  |  | Phase 4 | Digital health strategy exists and is fully aligned to country's UHC goals, but does not include metrics to assess the contribution of digital health towards UHC goals. |
|  |  | Phase 5 | Digital health strategy is fully aligned to the country's UHC goals and includes metrics to assess the contribution of digital health toward UHC targets. |
| Variable 9 | Indicator 6 -Public funding for digital health | Phase 1 | No budget line item for digital health available. |
|  |  | Phase 2 | Non-systematic budget allocated for digital health exists or are projects and/or system-based. |
|  |  | Phase 3 | A structured and systematic budget line item for digital health exists but is significantly insufficient (less than 50% of need) to meet the country's digital health needs. |
|  |  | Phase 4 | A structured and systematic budget line item for digital health exists but is moderately insufficient (above 50% of need) to meet the country's digital health needs. |
|  |  | Phase 5 | Structure and systematic budget line exists for digital health and is completely sufficient to meet the country's digital health needs. |
| Variable 10 | Indicator 6a -Private sector participation and investments in digital health | Phase 1 | The private sector do not visibly participate and invest in country's digital health activities. |
|  |  | Phase 2 | The private sector participation and investment in the country's digital health activities and investment is ad-hoc and limited. |
|  |  | Phase 3 | The private sector participation and investment in the country's digital health activities is systematic but does not meet the needs of the country. |
|  |  | Phase 4 | The private sector participation and investment in the country's digital health activities is systematic and meets the needs of the country. |
|  |  | Phase 5 | The private sector participates and invests in the country's digital health activities in an optimized environment. |

**Table 3: Variables applied to construct ACRI**

| **Variable** | **Pillar** | **Phases of maturity** | **Description** |
| --- | --- | --- | --- |
|  | **Legislation, Policy & Compliance** |  |  |
| Variable 11 | Indicator 7 -Legal Framework for Data Protection (Security/ Cybersecurity) | Phase 1 | There is no law on data security (across the full data lifecycle e.g. collection, processing, storage, transmission, use and destruction) that is relevant to digital health. |
|  |  | Phase 2 | There is a law on data security (across the full data lifecycle e.g. collection, processing, storage, transmission, use and destruction) that is relevant to digital health that has been proposed and is under review. |
|  |  | Phase 3 | There is a law on data security (across the full data lifecycle e.g. collection, processing, storage, transmission, use and destruction) that is relevant to digital health that has been passed, but has not yet been fully implemented. |
|  |  | Phase 4 | There is a law on data security (across the full data lifecycle e.g. collection, processing, storage, transmission, use and destruction), that is relevant to digital health that has been implemented, but not consistently enforced. |
|  |  | Phase 5 | There is a law on data security (across the full data lifecycle e.g. collection, processing, storage, transmission, use and destruction) that is relevant to digital health that has been implemented and enforced consistently. |
| Variable 12 | Indicator 8 -Laws or Regulations for privacy, consent, confidentiality and access to health information (Privacy) | Phase 1 | There is no law to protect individual privacy, governing ownership, consent, access and sharing of individually identifiable digital health data. |
|  |  | Phase 2 | There is a law to protect individual privacy, governing ownership, consent, access and sharing of individually identifiable digital health data that has been proposed and is under review. |
|  |  | Phase 3 | There is a law to protect individual privacy, governing ownership, consent, access and sharing of individually identifiable digital health data that has been passed, but not yet fully implemented. |
|  |  | Phase 4 | There is a law to protect individual privacy, governing ownership, access, consent, and sharing of individually identifiable digital health data that has been implemented, but not consistently enforced. |
|  |  | Phase 5 | There is a law to protect individual privacy, governing ownership, access, consent, and sharing of individually identifiable digital health data that has been implemented and is enforced consistently. Specific laws and protections are in place to protect the privacy of children and other vulnerable groups. |
| Variable 13 | Indicator 9 -Protocol for regulating or certifying devices and/or health services- including provisions for AI and algorithms (at higher stages of maturity) | Phase 1 | There are no protocols, policies, frameworks or accepted processes governing the clinical and patient care use of connected medical devices and health services (e.g. telemedicine, applications), particularly in relation to safety, data integrity and quality of care. |
|  |  | Phase 2 | Protocols, policies, frameworks or accepted processes governing the clinical and patient care use of connected medical devices and digital health services (e.g. telemedicine, applications), particularly in relation to safety, data integrity and quality of care have been proposed and are under review. |
|  |  | Phase 3 | Protocols, policies, frameworks or accepted processes governing the clinical and patient care use of connected medical devices and digital health services (e.g. telemedicine, applications), particularly in relation to safety, data integrity and quality of care have been passed, but are not fully implemented. |
|  |  | Phase 4 | Protocols, policies, frameworks or accepted processes governing the clinical and patient care use of connected medical devices and health services (e.g. telemedicine, applications), particularly in relation to safety, data integrity and quality of care --including provisions for AI and algorithms- have been implemented, but not consistently enforced. |
|  |  | Phase 5 | Protocols, policies, frameworks or accepted processes governing the clinical and patient care use of connected medical devices and health services (e.g. telemedicine, applications), particularly in relation to safety, data integrity and quality of care - including provisions for AI and algorithms- have been implemented and are enforced consistently. |
| Variable 14 | Indicator 9a -Protocol for regulating and certifying AI within health services | Phase 1 | There are no protocols, policies, frameworks, or accepted processes governing AI use in health services. |
|  |  | Phase 2 | Protocols, policies, frameworks, or accepted processes governing AI use in health service have been proposed and under review |
|  |  | Phase 3 | Protocols, policies, frameworks, or accepted processes governing AI use in health service have been passed but are not fully implemented. |
|  |  | Phase 4 | Protocols, policies, frameworks or accepted processes governing AI use in health service have been implemented but are not consistently enforced. |
|  |  | Phase 5 | Protocols, policies, frameworks, or accepted processes governing AI use in health service have been implemented and are enforced consistently. |
| Variable 15 | Indicator 10 -Cross-border data security and sharing | Phase 1 | There are no protocols, policies, frameworks or accepted processes in place to support secure cross-border data exchange and storage in support of public health goals while protecting individual privacy. |
|  |  | Phase 2 | Protocols, policies, frameworks or accepted processes for cross border data exchange and storage in support of public health goals while protecting individual privacy have been proposed and are under review. |
|  |  | Phase 3 | Protocols, policies, frameworks or accepted processes for cross border data exchange and storage in support of public health goals while protecting individual privacy have been passed, but are not fully implemented. |
|  |  | Phase 4 | Protocols, policies, frameworks or accepted processes for cross border data exchange and storage in support of public health goals while protecting individual privacy have been implemented, but not consistently enforced. |
|  |  | Phase 5 | Protocols, policies, frameworks or accepted processes for cross border data exchange and storage in support of public health goals while protecting individual privacy have been implemented and enforced consistently. |
|  | **Workforce** |  |  |
| Variable 16 | Indicator 11 -Digital health integrated in health and related professional pre-service training (prior to deployment) | Phase 1 | There is no digital health curriculum for health professionals as part of pre-service training requirements. |
|  |  | Phase 2 | Digital health curriculum proposed and under review as part of pre-service training requirements. |
|  |  | Phase 3 | Digital health curriculum implementation underway covering an estimated <50% of health professionals in pre-service training. |
|  |  | Phase 4 | Digital health taught in relevant institutions with an estimated 50-75% health professionals receiving pre-service training. |
|  |  | Phase 5 | Digital health taught in relevant institutions with >75% of health professionals receiving pre-service training. |
| Variable 17 | Indicator 12 -Digital health integrated in health and related professional in-service training (after to deployment) | Phase 1 | There is no digital health curriculum as part of in-service (continuing education) training for health professionals in the workforce. |
|  |  | Phase 2 | Digital health curriculum proposed and under review as part of in-service (continuing education) training for health professionals in the workforce. |
|  |  | Phase 3 | Digital health curriculum is implemented as part of in-service (continuing education) training for <50% health professionals in the workforce. |
|  |  | Phase 4 | Digital health curriculum is implemented as part of in-service (continuing education) training for 50-75% health professionals in the workforce. |
|  |  | Phase 5 | Digital health curriculum is implemented as part of in-service (continuing education) training for >75% health professionals in the workforce. |
| Variable 18 | Indicator 13 -Training of digital health workforce | Phase 1 | There is no training available for digital health workforce in the country. |
|  |  | Phase 2 | Digital health workforce needs assessed, gaps identified and training options under development. |
|  |  | Phase 3 | Professional training is available, but graduates are not yet deployed. |
|  |  | Phase 4 | Trained digital health professionals available and deployed, but essential personnel gaps remain. |
|  |  | Phase 5 | Sufficient numbers of trained digital health professionals available to support national digital health needs. |
| Variable 19 | Indicator 14 -Maturity of public sector digital health professional careers | Phase 1 | No workforce strategy, policy, or guide that recognizes digital health is in place. Distribution of digital health workforce is ad hoc. |
|  |  | Phase 2 | A national needs assessment shows the number and types of skills needed to support digital health with an explicit focus on training cadres of female health workers. |
|  |  | Phase 3 | Digital health staff roles and responsibilities are mapped to the government's workforce and career schemes and 25-50% of needed public sector digital health workforce in place. |
|  |  | Phase 4 | An HR policy and strategic plan exists that identifies skills and functions needed to support digital health with an explicit focus on training cadres of female health workers and an estimated 50-75% of public sector digital health workforce in place. |
|  |  | Phase 5 | A long-term plan is in place to grow and sustain staff with the skills needed to sustain digital health at national and subnational levels with an explicit focus on training cadres of female health workers with an estimated >75% of positions needed filled. Performance management systems are in place to ensure growth and sustainability of the digital health workforce with sufficient supply to meet digital health needs and little staff turnover. |
